# Reversal of MK-801-Induced Disruptions in Social Interactions and Working Memory with Simultaneous Administration of LY487379 and VU152100 in Mice

**DOI:** 10.3390/ijms20112781

**Published:** 2019-06-06

**Authors:** Paulina Cieślik, Adrianna Radulska, Iwona Pelikant-Małecka, Agata Płoska, Leszek Kalinowski, Joanna M Wierońska

**Affiliations:** 1 Maj Institute of Pharmacology, Polish Academy of Sciences, 31-343 Kraków, Poland; cieslik@if-pan.krakow.pl; 2 Department of Medical Laboratory Diagnostics-Biobank, Medical University of Gdansk, 80-211 Gdansk, Poland; adrianna.radulska@gumed.edu.pl (A.R.); iwona.pelikant-malecka@gumed.edu.pl (I.P.-M.); agata.ploska@gumed.edu.pl (A.P.); leszek.kalinowski@gumed.edu.pl (L.K.); 3 Biobanking and Biomolecular Resources Research Infrastructure Poland (BBMRI.PL), 80-211 Gdansk, Poland

**Keywords:** schizophrenia, metabotropic glutamate receptors, muscarinic receptors, negative and cognitive symptoms

## Abstract

Negative and cognitive symptoms of schizophrenia contribute to an impaired social and professional life for schizophrenic patients, and in most cases, these symptoms are treatment resistant. Therefore, identification of new treatment strategies is sorely needed. Metabotropic glutamate receptors (mGlu) and muscarinic (M) receptors for acetylcholine have been considered promising targets for novel antipsychotics. Among them, mGlu_2_ and M_4_ subtypes seem to be of particular importance. In the present study, the effect of mutual activation of mGlu_2_ and M_4_ receptors was assessed in MK-801-based animal models of negative and cognitive symptoms of schizophrenia, that is, social interaction and novel object recognition tests. Low sub-effective doses of LY487379 (0.5 mg/kg), a positive allosteric activator of the mGlu_2_ receptor, and VU152100 (0.25−0.5 mg/kg), a positive allosteric modulator of the M_4_ receptor, were simultaneously administered in the aforementioned tests. Combined administration of these compounds prevented MK-801-induced disturbances in social interactions and object recognition when acutely administered 30 min before MK-801. Prolonged (7 days) administration of these compounds resulted in the loss of effectiveness in preventing MK-801-induced disruptions in the novel object recognition test but not in the social interaction test. In the next set of experiments, MK-801 (0.3 mg/kg) was administered for seven consecutive days, and the activity of the compounds was investigated on day eight, during which time MK-801 was not administered. In this model, based on prolonged MK-801 administration, the effectiveness of the compounds to treat MK-801-induced disruptions was evident at low doses which were ineffective in preventing the behavioural disturbances induced by an acute MK-801 injection. Combined administration of the compounds did not exert better efficacy than each compound given alone. Pharmacokinetic analysis confirmed a lack of possible drug–drug interactions after combined administration of LY487379 and VU152100. Our data show that modulation of M_4_ and mGlu_2_ receptors may potentially be beneficial in the treatment of negative and cognitive symptoms of schizophrenia.

## 1. Introduction

The mechanism of action of currently used antipsychotic drugs is predominantly dopamine-based; therefore, the use of these drugs is burdened with a high risk of inducing adverse effects related to the blockade of D_2_ receptors in the striatum or hypothalamus [1]. The most troublesome are extrapyramidal side effects, increased prolactin levels, impaired motor coordination and increased body weight [2]. In most cases, development of these adverse effects contributes to worsening quality of life and results in cessation of treatment.

In addition to the high risk of inducing adverse effects, the efficacy of currently used neuroleptics remains unsatisfactory. The majority of drugs, particularly typical neuroleptics, effectively restore positive symptoms of schizophrenia but do little for negative or cognitive symptoms [3]. Much more effective, and slightly better tolerated, are atypical drugs that have an affinity not only toward D_2_ receptors, in contrast to typical antipsychotics, but also toward other receptors, including serotonergic, adrenergic or histamine receptors [4]. This multidirectional mechanism of action of atypical neuroleptics probably accounts for their improved efficacy compared to typical drugs and may convey better therapeutic activity of the combined treatment. 

Our previous studies indicated the benefits resulting from simultaneous activation of at least two well-defined receptors (e.g., mGlu_5_-GABA_B_, mGlu_4_-5-HT_1A_; see [5,6,7]). In our studies, we followed the glutamatergic theory of schizophrenia, according to which increased release of glutamate in the cortex is responsible for schizophrenia arousal [8]. This glutamate efflux results from inhibition of the NMDA (N-methyl-D-aspartate) receptor by non-competitive channel blockers, such as MK-801 or PCP [9]. Consequently, pharmacological interventions aimed at decreasing glutamate release seem to be most adequate for the pathophysiology of this disease. Activation of inhibitory presynaptic receptors expressed on glutamatergic nerve terminals directly decreased glutamate release [10]. The indirect inhibition of excessive glutamate transmission is also possible, for example, via cholinergic innervation of glutamatergic pyramidal neurons [11,12].

Among the receptors involved in presynaptic regulation of glutamate release are mGlu_2_ and M_4_ receptors, linked to G_i/0_-dependent signalling [11,12,13,14]. Their stimulation leads to the induction of inhibitory processes inside neurons and, consequently, inhibition of neurotransmitter release [11,12,13,14].

Both receptors are expressed in the cerebral cortex, striatum and hippocampus, which are brain regions of particular importance in schizophrenia [15,16], and disturbances in the expression of M_4_ receptor in schizophrenia patients have been confirmed by some post mortem studies [17]. A growing body of preclinical studies shows that mGlu_2_ and M_4_ receptor stimulation reverses deficits observed in animal models of schizophrenia [18,19,20,21,22,23,24,25]. mGlu_2_ ligands were also studied in clinical trials with mixed results (initial positive data on antipsychotic activity of mGlu_2_ ligands in schizophrenic patients were contradicted by subsequent Phase III studies) [26,27], and there is no available clinical data concerning selective M_4_ ligands. A clinical trial involving xanomeline, a non-selective M_1_/M_4_-preferring agonist, reported that modulation of muscarinic receptors is beneficial [28]. Importantly, M_4_ receptors are predominantly expressed in the central nervous system, and in contrast to M_2_ or M_3_ muscarinic receptor subtypes, their stimulation is less burdened by adverse effects due to activation of receptors expressed in peripheral tissues. 

In our previous research it was shown that positive allosteric modulator (PAM) of M_4_ receptor, VU152100, alone and in combined administration with the orthosteric agonist of mGlu_4_ receptor, LSP4-2022, was effective in animal models of schizophrenia, such as MK-801- or amphetamine-induced hyperactivity, DOI (2,5-dimethoxy-4-iodoamphetamine)-induced head twitches, modified forced swim test, MK-801-induced disruptions in social interactions and novel object recognition test [18]. 

This study is a follow-up study in which the effect of simultaneous activation of mGlu_2_ and M_4_ receptors was investigated in the social interaction and novel object recognition tests, animal models of negative and cognitive symptoms of schizophrenia.

In this set of experiments, we used three different schemes of drug administration to establish whether the investigated ligands prevent and treat schizophrenia symptoms. To determine whether tolerance develops, activity of the compounds was investigated after their chronic administration. Pharmacokinetic experiments were performed to exclude possible drug–drug interactions that could influence the results obtained in behavioural tests.

## 2. Results

### 2.1. Treatment Regimens

**Figure 1:**
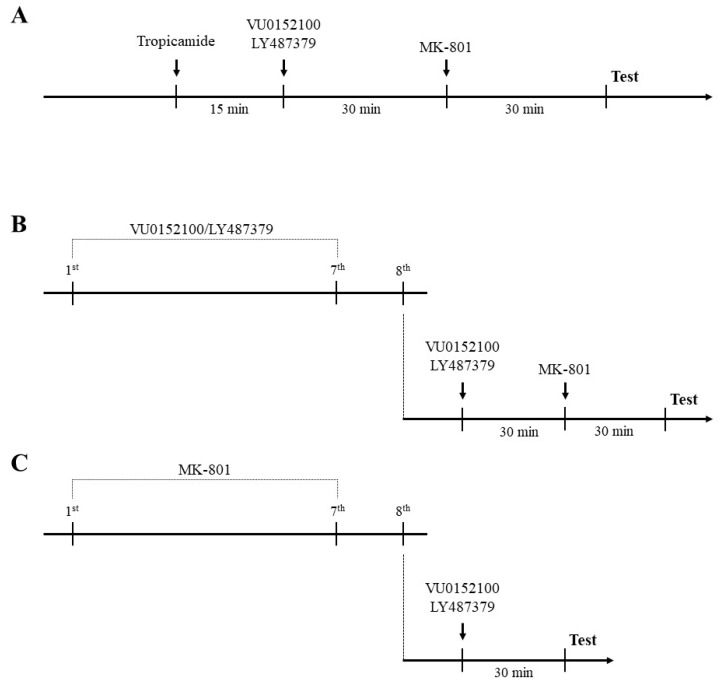
Schematic representation of the experiments performed in this study. (**A**) Acute administration of investigated compounds and MK-801. (**B**) Prolonged administration of investigated compounds and acute administration of MK-801. (**C**) Prolonged administration of MK-801 and acute administration of investigated compounds.

Drugs were administered by three different schedules: (1) Acute administration of compounds 45 min (Tropicamide—M_4_-preffering antagonist) or 30 min (VU152100, LY487379) before MK-801 (also administered once, 30 min before the test or T_1_ in novel object recognition test); (2) prolonged administration (once per day for 7 consecutive days) of compounds (VU152100, LY487379) with the last administration on day 8, 30 min before MK-801 (and the test 30 min after MK-801 administration); and (3) prolonged administration of MK-801 (0.3 mg/kg, once per day for 7 days) with compounds administered acutely 24 h after the last MK-801 administration and 30 min before the test. Schedules of administration are presented graphically in Figure 1. The sub-effective doses of VU152100 for each test (0.25 mg/kg for novel object recognition and 0.5 mg/kg for social interaction) were chosen based on our previous research (see [18]). Different vehicles were used throughout the study: (1) 0.9% NaCl, (2) 10% Tween 80 or (3) 10% Tween 80 containing 2% DMSO. Vehicles were administered to all animals (e.g., control mice, MK-801-treated mice) in the same schedule as drugs were administered to the drug-treated appropriate groups of mice. Neither solvent exerted independent effects on animal behaviour.

### 2.2. Novel Object Recognition

#### 2.2.1. Effectivity of Acutely Administered Compounds in Animals after Acute Treatment with MK-801

**Figure 2:**
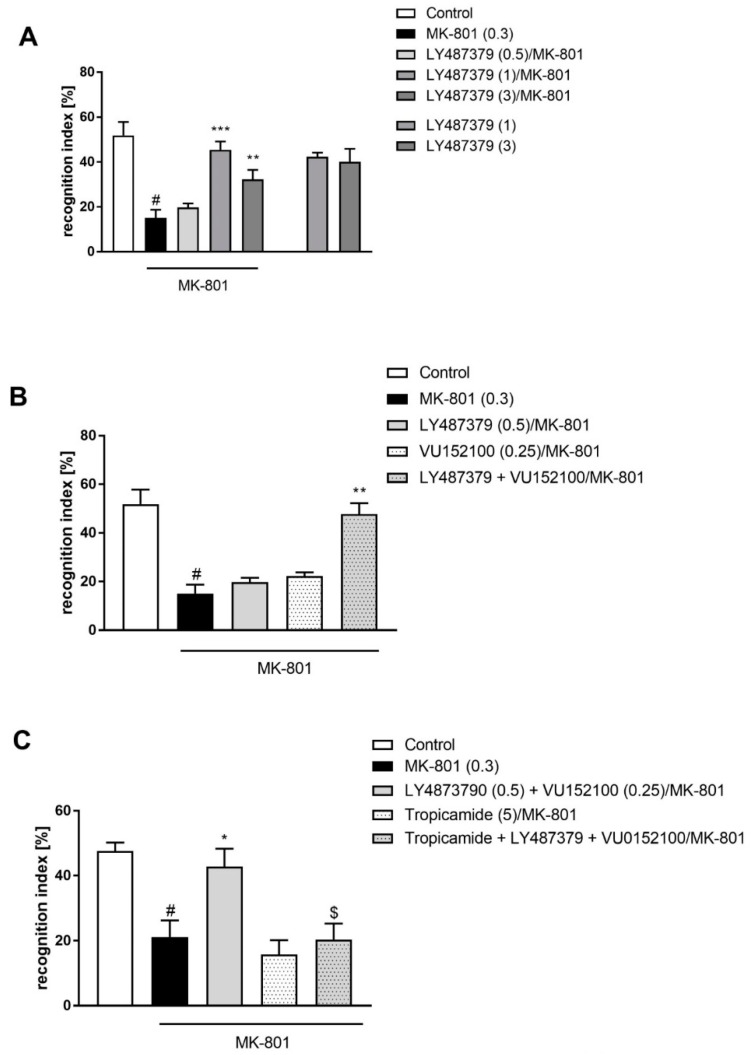
Novel object recognition test in mice. Effectivity of LY487379 (**A**), combined administration of sub-effective doses of LY487379 and VU152100 (**B**), and the blockade of combination’s effect by M_4_ receptor antagonist Tropicamide (**C**). Compounds and MK-801 were administered acutely, as shown in Figure 1. Investigated doses are indicated in parentheses. Bars represent the means ± SEM. N = 8–10/group. # *p* < 0.001 versus the control group; * *p* < 0.05 and ** *p* < 0.01 versus the MK-801-treated group; ^$^ *p* < 0.0001 versus the LY487379 + VU152100 group.

LY487379 was administered at 0.5–3 mg/kg doses, and the best effectivity was observed at doses of 1 and 3 mg/kg (F_(3.34)_ = 14.84; *p* < 0.0001), which prevented MK-801-induced disruptions in object recognition. A dose of 0.5 mg/kg was ineffective (Figure 2A). The compound had no effect on learning abilities when given alone.

Simultaneous administration of ineffective doses of LY487379 (0.5 mg/kg) with VU152100 (0.25 mg/kg) 30 min before MK-801 induced the same effect as each compound alone at effective doses (F_(1.32)_ = 10.88) (Figure 2B).

The effect of combined administration of LY487379 (0.5 mg/kg) and VU152100 (0.25 mg/kg) was blocked by Tropicamide given at 5 mg/kg 15 min before the compounds and 45 min before MK-801. Two-way ANOVA revealed a significant effect of this interaction: F_(1.15)_ = 18.67; *p* < 0.0001 (Figure 2C). 

#### 2.2.2. Effectivity of Prolonged Administration of Compounds in Animals Acutely Treated with MK-801

**Figure 3:**
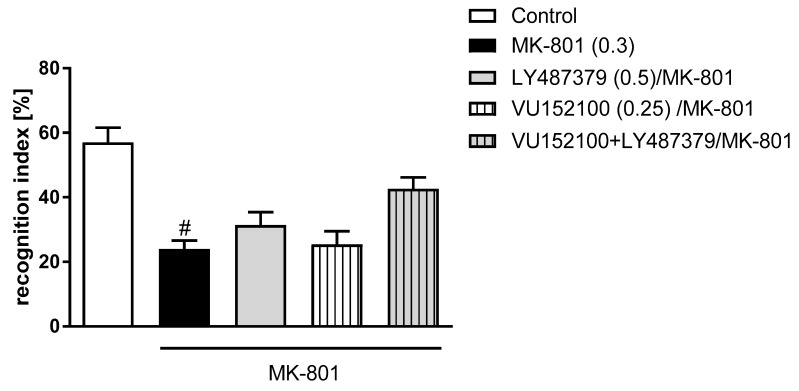
Novel object recognition test in mice after chronic administration (7 days) of investigated compounds in acute MK-801 model. Investigated doses are indicated in parentheses. Bars represent the means ± SEM. N = 8–10/group. ^#^ *p* < 0.001 versus the control group.

The investigated compounds did not prevent the MK-801-induced deficits after prolonged administration neither when administered alone, nor in the combinations of their sub-effective doses (Figure 3).

#### 2.2.3. Effectivity of Acutely Administered Compounds in Animals Repeatedly Treated with MK-801

**Figure 4:**
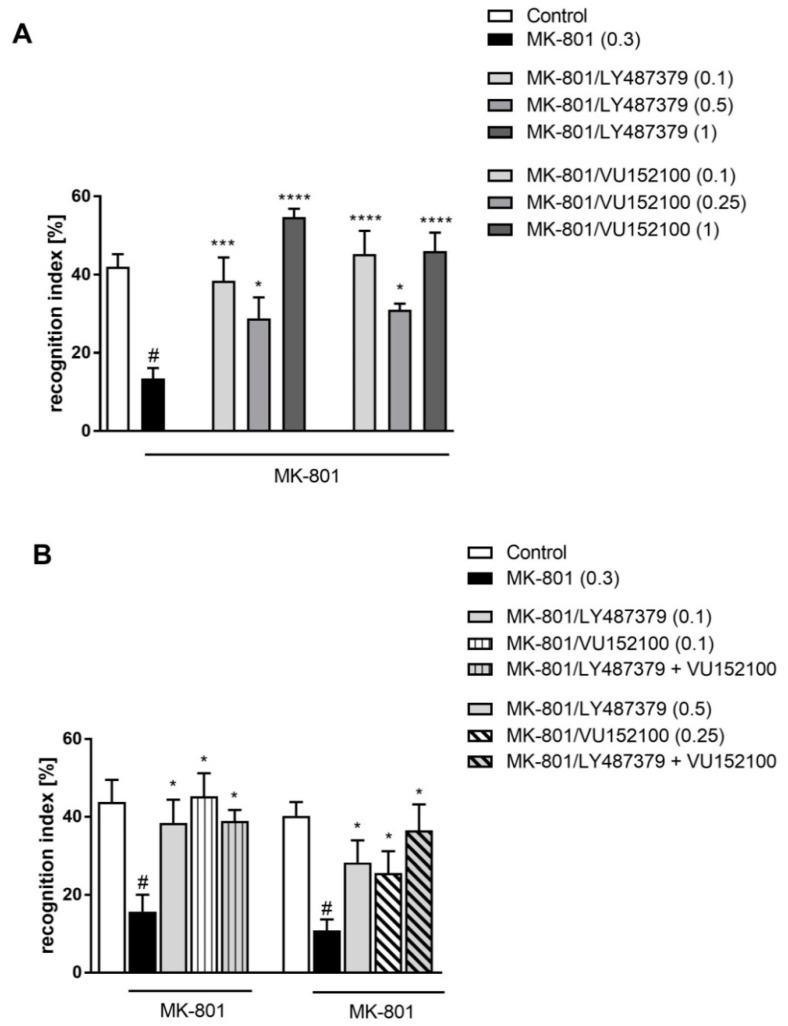
Novel object recognition test in mice after chronic administration (7 days) of MK-801. Effectivity of LY487379 and VU152100 administered alone (**A**), and combined administration of low doses of LY487379 and VU152100 (**B**). Compounds were administered acutely 30 min before the test. Investigated doses are indicated in parentheses. Bars represent the means ± SEM. N = 8–10/group. # *p* < 0.001 versus the control group; * *p* < 0.05 and *** *p* < 0.001 and **** *p* < 0.0001 versus the MK-801-treated group.

Administration of MK-801 (0.3 m/kg) for 7 days induced deficits in the novel object recognition paradigm observed 24 h after the last administration (Figure 4). Compounds treated the MK-801-induced deficits at all administered doses: LY487379 at doses 0.1, 0.5 and 1 mg/kg and VU152100 at doses 0.1, 0.25 and 1 mg/kg. One-way ANOVA revealed a significant effect of these compounds, F_(3.26)_ = 21.87; *p* < 0.0001 and F_(3.24)_ = 16.68; *p* < 0.0001, respectively (Figure 4A). Concomitant administration of compounds did not enhance their action in this paradigm (Figure 4B). 

### 2.3. Social Interaction Test

#### 2.3.1. Effectivity of Acutely Administered Compounds in Animals after Acute Treatment with MK-801

**Figure 5:**
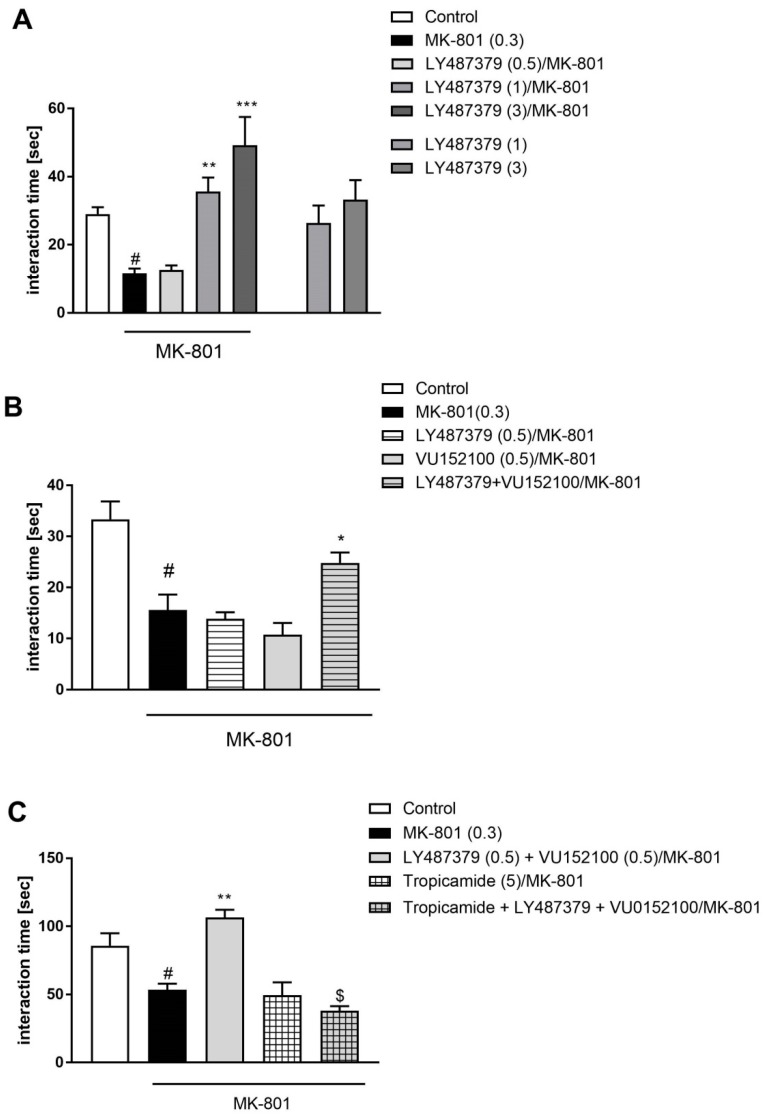
Social interaction test in mice. Effectivity of LY487379 (**A**), combined administration of sub-effective doses of LY487379 and VU152100 (**B**), and the blockade of the combination’s effect by M_4_ receptor antagonist Tropicamide (**C**). Compounds and MK-801 were administered acutely as shown on Figure 1. The investigated doses are indicated in parentheses. Bars represent the means ± SEM. N = 8–10/group. # *p* < 0.001 versus the control group; * *p* < 0.05, ** *p* < 0.01 and *** *p* < 0.001 versus the MK-801-treated group; ^$^ *p* < 0.0001 versus the LY487379 + VU152100 group.

LY487379 was administered at 0.5–3 mg/kg doses, and prevented the MK-801-induced deficits at doses of 1 and 3 mg/kg (F_(3.23)_ = 7.13; *p* < 0.0015). The 0.5 mg/kg dose was ineffective (Figure 5A). The compound had no effect on social behaviour when given alone.

Simultaneous administration of ineffective doses of LY487379 (0.5 mg/kg) and VU152100 (0.5 mg/kg) 30 min before MK-801 also prevented MK-801-induced deficits (Figure 5B), as calculated by two-way ANOVA (F_(1.23)_ = 9.89; *p* < 0.005).

The effect of combined administration of LY487379 (0.5 mg/kg) and VU152100 (0.5 mg/kg) was blocked by Tropicamide, given at a dose of 5 mg/kg 15 min before the compounds. Two-way ANOVA revealed a significant effect of this interaction: F_(1.20)_ = 30.68; *p* < 0.0001 (Figure 5C). 

#### 2.3.2. Effectivity of Prolonged Administration of Compounds in Animals Acutely Treated with MK-801

**Figure 6:**
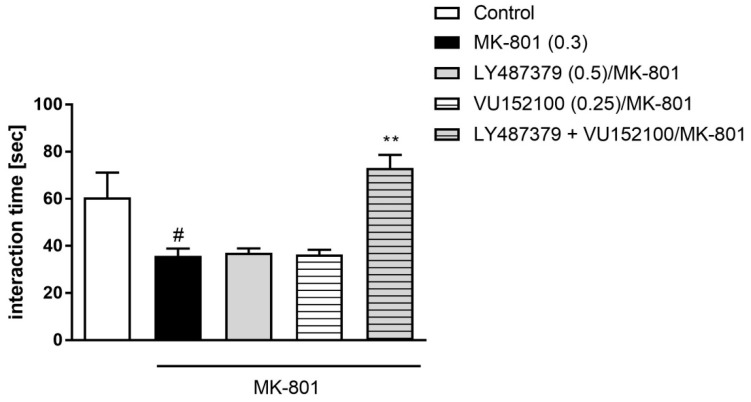
Social interaction test in mice after chronic administration (7 days) of investigated compounds in acute MK-801 model. Investigated doses are indicated in parentheses. Bars represent the means ± SEM. N = 8–10 pairs/group. # *p* <0.001 versus the control group and ** *p* < 0.01 versus LY487379 or VU152100.

The effectiveness of compounds to prevent MK-801-induced disruptions after their prolonged administration was the same as after acute administration, as calculated by two-way ANOVA (F_(1.21)_ = 11.9) (Figure 6).

#### 2.3.3. Effectivity of Acute Administration of Compounds in Animals Repeatedly Treated with MK-801

**Figure 7:**
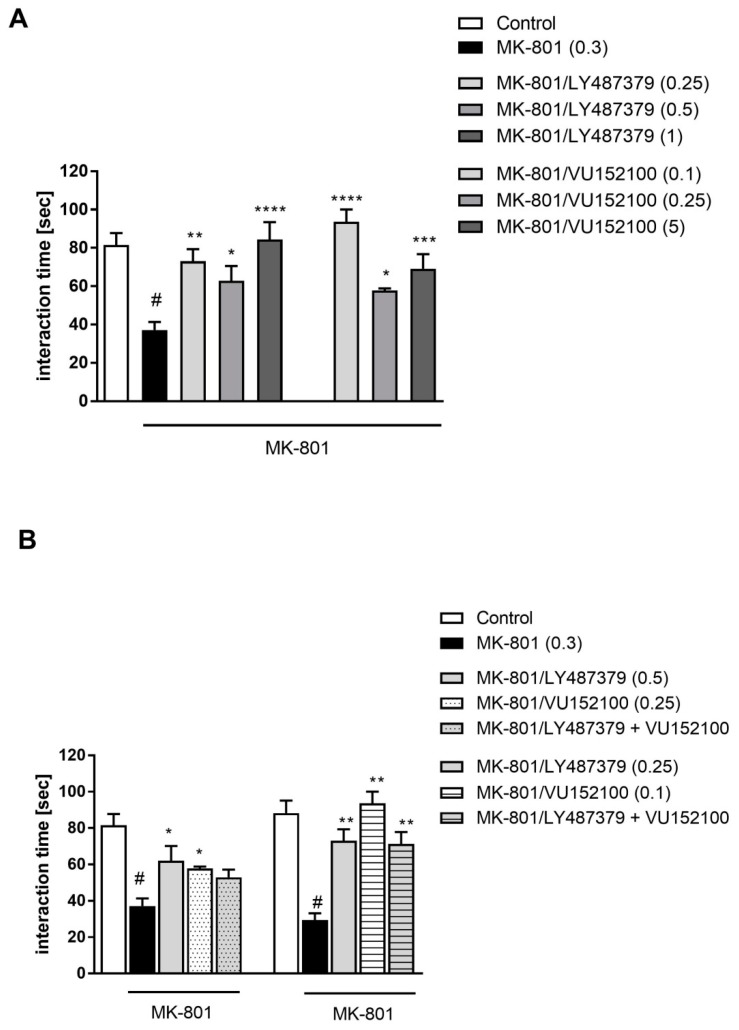
Social interaction test in mice after chronic administration (7 days) of MK-801. Effectivity of LY487379 and VU152100 administered alone (**A**), and combined administration of low doses of LY487379 and VU152100 (**B**) to treat MK-801-induced dysfunctions. Compounds were administered acutely 30 min before the test. Investigated doses are indicated in parentheses. Bars represent the means ± SEM. N = 8–10 pairs/group. # *p* < 0.001 versus the control group; * *p* < 0.05, ** *p* < 0.01, *** *p* < 0.001 and **** *p* < 0.0001 versus the MK-801-treated group.

Administration of MK-801 (0.3 mg/kg) for 7 days induced deficits in social interactions, observed 24 h after the last injection (Figure 7). LY487379 was administered at 0.25, 0.5 and 1 mg/kg doses, and VU152100 was administered at 0.1, 0.25 and 5 mg/kg doses. The compounds treated MK-801-induced deficits at all administered doses. One-way ANOVA revealed a significant effect of the compounds, F_(3.21)_ = 10.32; *p* < 0.0002 and F_(3.22)_ = 20.53; *p* < 0.0001, respectively (Figure 7A). Simultaneous administration of low doses of both compounds did not enhance their individual effectiveness to treat MK-801-induced deficits (Figure 7B).

### 2.4. Evaluation of the Drug Concentration in Plasma and Brain

We separately evaluated drug exposure levels of each compound at the highest dose and the combination of LY487379 and VU152100, each at a sub-effective dose, in the plasma and brain. Concentrations of the compounds were calculated both in mice acutely treated with MK-801 and in mice that received prolonged (7 days) MK-801 administration. Comparing the brain penetration abilities of both compounds when given alone at top doses and when administered in combined sub-effective doses to mice acutely treated with MK-801, a slight decrease in brain penetration for LY487379 (brain/plasma ratios 1.28 vs. 1.07) and slightly better brain penetration for VU152100 (0.12 vs. 0.17) were observed. In mice chronically administered with MK-801, no significant changes in the brain/plasma ratios were observed when drugs were administered alone at the highest doses (compared to mice acutely treated with MK-801), while an improvement in the brain/plasma ratios was observed for combined low doses of LY487379 (1.38, ~30% increase of compound concentration in the brain) and VU152100 (0.29, ~70% increase of compound concentration in the brain) compared to animals with single MK-801 administration (Table 1). 

## 3. Discussion

In the present study, we evaluated the impact of simultaneous administration of mGlu_2_ and M_4_ receptor activators in animal models of schizophrenia. We used LY487379 and VU152100, known and described previously as effective antipsychotics in animal models [21,22,29]. Herein, we focused on simultaneous administration of these ligands in novel object recognition and social interaction tests, assessing cognitive and negative symptoms in animal models of schizophrenia. As mentioned in the introduction, those symptoms do not respond well to presently used antipsychotic medications and contribute to worsening quality of life.

The investigated compounds were tested in schizophrenia models induced by acute and prolonged MK-801 administration to evaluate drug activity with different schedules. 

Acute administration of MK-801 is a well-established model of schizophrenia, with high predictive validity for investigating the antipsychotic-like activity of compounds. This model is widely used in a variety of studies, including those performed in our laboratory (for example, [7,18]). Using this model, we showed antipsychotic-like activity of VU152100 in social interaction and in novel object recognition tests [18,30]. Data concerning the antipsychotic-like activity of mGlu_2_ activators can be found elsewhere [23,25,31,32,33], but despite the variety of different reports concerning LY487379 in animal models, its activity in social interaction and novel object recognition is poorly investigated so far, and no dose-response studies have been described. Therefore, we also investigated the activity of this compound alone. In both tests, the compound induced clear dose-dependent efficacy preventing MK-801-induced disruptions, confirming its antipsychotic-like effects. Subsequently, LY487379 was administered simultaneously with VU152100, both compounds at low, sub-effective doses. This concomitant administration produced the same effect on MK-801-induced dysfunctions in novel object recognition and social interactions as each compound alone at their effective doses. The effect of this combination was shown to be receptor-specific, as the efficacy of simultaneous administration of these compounds was blocked by the M_4_ receptor antagonist Tropicamide. Additionally, any potential drug–drug interactions that could influence behavioural results were excluded in pharmacokinetic studies, which were performed according to earlier studies [30,34]. Comparing the brain/blood ratios of these compounds in high versus low doses, no drug–drug interactions that significantly contributed to behavioural results were observed.

Subsequently, sub-effective doses of the drugs alone and in combination were administered for seven consecutive days to evaluate if prolonged administration of compounds induces changes in animal responses based on their action, in particular, development of tolerance. The effectiveness to prevent MK-801-induced deficits by combined administration of these compounds disappeared in the novel object recognition test but not in the social interaction test. This finding indicates superior activity of these compounds towards negative symptoms of schizophrenia. To date, it is understood that the development of tolerance may happen after chronic administration of mGlu_2/3_ activators and was described earlier for LY379268 in PCP-induced hyperactivity [22]. 

The effect of the compounds observed in an acute MK-801 model of schizophrenia included only prevention of deficits caused by MK-801 (Figure 2, Figure 3, Figure 5, and Figure 6). The drugs were not administered after MK-801 to see if they were potent to treat MK-801-induced deficits. Such experiments were performed in the second part of the study, where the efficacy of both drugs was evaluated in animal models of negative and cognitive symptoms of schizophrenia induced by chronic administration of MK-801 (Figure 4 and Figure 7). Chronic administration of NMDA channel blockers (MK-801 or PCP) is often used in studies on the antipsychotic action of drugs [35]. There is no clear evidence for how long psychoactive substances should be administered to obtain schizophrenia-like deficits in animal models. Different schedules of administration are used, and the range for drug administration varies from five to 15 days [36,37,38,39,40]. The last administration of compounds occurs either 24 h before or on the test day and is dependent on laboratory standards [41,42,43].

In the present study, an administration schedule of seven days of MK-801 administration was used and tests were performed 24 h after the last injection. In both the social interaction and novel object recognition tests, the observed dysfunctions were similar to those observed after a single MK-801 administration.

In this schedule, investigated compounds were administered 30 min before the test. In contrast to the acute MK-801 administration schedule, the aim of these experiments was to evaluate whether the investigated compounds treat dysfunctions induced by chronic administration of MK-801. 

Prolonged administration of MK-801 resulted in changes of behavioural responses of mice to mGlu_2_ and M_4_ activators. The investigated doses of both compounds exerted much better effectiveness in treating MK-801-induced deficits than to prevent them in the acute MK-801 model. This may indicate a change in blood–brain barrier permeability or an increase in sensitivity or expression of mGlu_2_ and M_4_ receptors after prolonged MK-801 treatment. Although these aspects would be worth investigating, these issues are beyond the scope of this project and remain to be investigated. Pharmacokinetic analyses were performed to determine whether the brain/plasma ratios of tested compounds changed in animals chronically treated with MK-801 compared to mice acutely treated with MK-801. Compounds were administered at the same schedule. Prolonged administration of MK-801 did not change brain/plasma ratios for the top doses of both compounds. After their combined administration in sub-effective doses a 30% increase in brain concentration of LY487379 and a 70% increase in brain concentration of VU152100 were observed in mice repeatedly treated with MK-801 comparing to animals with acute MK-801 administration. 

No enhancement of compound effectivity to treat schizophrenia-related behavioural impairments was observed when LY487379 and VU152100 were administered simultaneously at low doses in a prolonged MK-801 model.

Both investigated ligands activate G protein coupled receptors and stimulate G_i/0_ proteins linked with adenylyl cyclase activity, exerting inhibitory activity in neurons [44]. mGlu_2_ and M_4_ receptors are expressed on glutamatergic terminals that innervate glutamatergic neurons [14,45]. Their stimulation leads to inhibition of glutamate release, resulting in antipsychotic action in animal models [8,12,18]. Additionally, M_4_ receptors are also expressed presynaptically on cholinergic interneurons and control the activity of entorhinal and perirhinal cortex projections, thus contributing to an improvement in cognitive functions [46,47]. Although these compounds are effective when given alone, their combined administration at sub-effective doses could be safer and less burdened with the risk of inducing adverse effects. Compounds administered in combination may supplement each other’s action and thus be more effective in patients with dysfunction or reduced expression of one type of receptor. As this treatment omits the inhibition of D_2_ receptors, it may be speculated that the compounds could be especially dedicated to so-called normodopaminergic subpopulation of schizophrenic patients resistant to dopamine-based drugs [48]. 

Treatment of negative and cognitive schizophrenia symptoms based on combined activation of mGlu_2_/M_4_ receptors creates new possibilities for the use of mGlu_2_ receptor ligands, which have been considered novel psychotropic drugs for years, exhibiting only slight success. After strong expectations and high hopes from initial promising results in clinical trials [26], the enthusiasm for mGlu_2_ ligands was dampened due to subsequent discouraging effects in humans [49]. One of the reasons that mGlu_2_ activators failed in the clinic is that their activity was tested in patients with predominantly positive symptoms who were previously treated with a variety of antipsychotic medication. Although this could be important, as indicated in several papers [50,51], the genetic susceptibility of patients was not taken into consideration. 

Taken together, we propose simultaneous stimulation of mGlu_2_/M_4_ receptors as a novel target for negative and cognitive symptoms of schizophrenia.

## 4. Materials and Methods

### 4.1. Animals and Housing

Male CD1 mice (Charles River, Germany) weighing 20–25 g at the time of arrival were used in behavioural experiments. Animals were kept in standard laboratory cages T3 (265 × 420 × 180 mm, with an area of 825 cm^2^), grouped at 10 mice per cage. Animals were kept under standard laboratory conditions (12:12 light:dark cycle, 21–22 °C) with free access to food and water. Animal welfare has been regularly controlled by a veterinarian and animal welfare committee. After 3–4 weeks of acclimatization and handling, the experiments began. Experimental groups consisted of 8 to 10 animals, depending on the procedure. Drugs were administered intraperitoneally (i.p.) at a volume of 10 mL/kg. Experimental assessments were performed by an observer who was blinded to treatment conditions. All procedures described herein were conducted in accordance with the European Communities Council Directive of 22 September 2010 (2010/63/EU) and Polish legislation acts concerning animal experimentation and were approved by the II Local Ethics Committee by the Institute of Pharmacology, Polish Academy of Sciences in Krakow (18/2019, 21.01.2019).

### 4.2. Drugs

MK-801, VU152100 (M4 PAM), LY487379 (mGlu2 PAM) and Tropicamide (M4-preferring muscarinic receptor antagonist) were purchased from Tocris Bioscience, Bristol, UK. DMSO and Tween 80 were purchased from Sigma-Aldrich, Schellendorf, Germany. MK-801 was dissolved in 0.9% NaCl, and VU152100 was dissolved in 10% Tween 80. LY487379 solution was prepared from a DMSO-containing stock by adjusting it with 0.9% NaCl to the proper volume (final DMSO concentration: <0.2%). Tropicamide was dissolved in a small amount of DMSO (final concentration: 2%) and then adjusted with 10% Tween 80 to the proper volume as described previously [30]. Vehicle-treated animals received appropriate solvents. Vehicle was administered to animals in any case when drug administration was omitted (e.g., control or MK-801-treated groups).

### 4.3. Novel Object Recognition Test

This procedure was adapted from Nilsson et al. [52] and performed as described in a variety of our previous papers [7,53]. Habituation, training and test trials were performed in a black plastic rectangular arena (40 × 30 × 35 cm) illuminated with a light intensity of 335 lux. During the habituation trial (2 consecutive days), each animal was allowed to explore the arena for 10 min. The next day, during the training trial (T_1_), mice were placed in the arena and were presented with two identical objects (red glass cylinder; 6.5 cm in diameter and 4.5 cm high) for 5 min. After 1 h, animals were placed back into the arena for a 5 min test trial, during which one of the previously presented familiar objects was replaced with a novel object (a transparent glass elongated sphere-like object with an orange cap; 5.5 cm in diameter and 8.5 cm high). Time spent exploring (i.e., sniffing or touching) the familiar (T_familiar_) and novel (T_novel_) objects was measured by a trained observer, and the recognition index [%] was calculated for each mouse [(T_novel_ – T_familiar_)/(T_familiar_ + T_novel_)] * 100.

### 4.4. Social Interaction Test

This procedure was adapted from de Moura Linck et al. [54] and performed as described in our previous papers [7,53]. Habituation and test trials were performed in a black plastic rectangular arena (40 × 30 × 35 cm) illuminated with a light intensity of 335 lux. During the habituation trial (2 consecutive days), each animal was allowed to explore the arena for 10 min. The test trial was conducted the following day, during which body-weight-matched mice (± 10% difference) from two different cages were placed into the arena for 10 min. Social interactions between the two mice were determined based on the total time spent participating in social behaviours, such as sniffing, genital investigation and chasing.

### 4.5. Evaluation of Drug Concentration in Plasma and Brain

This experiment was performed according to the methods described by Bridges et al. [34] and according to our previous experiments [30]. Brains and plasma were collected from acutely or repeatedly MK-801-treated mice that received a sub-active combination of VU152100 and LY487379 or the highest dose of either substance. Brains were removed, washed in 0.9% saline and frozen on dry ice. Blood was collected in EDTA-containing tubes (25 µL, 5%), and plasma was separated by centrifugation at 2000 rpm for 15 min at 4 °C and stored at −80 °C until analysis. 

On the day of analysis, frozen whole mouse brains were weighed and homogenized in 1:2 (*w*/*v*) volumes of ice-cold acetonitrile containing 0.1% FA and internal standard mix. Samples were homogenized using a Bio-Gen PRO200 homogenizer 220 v at minimum speed for 1 min, vortexed, and kept on ice for 20 min, followed by centrifugation at 14,000 rpm for 20 min at 4°C. Finally, 50 µL of supernatant was diluted with 0.1% FA in water (1:1, *v*/*v*) and analysed by HPLC/MS/MS.

Sample extraction of plasma (20 µL) was performed after spiking with an internal standard mix by protein precipitation using three volumes of ice-cold acetonitrile containing 0.1% formic acid. The extract was vortexed, kept on ice for 20 min and then centrifuged at 14,000 rpm for 20 min at 4 °C. The supernatant was diluted with 0.1% FA in water (1:1, *v*/*v*) and analysed by means of HPLC/MS/MS using a QTRAP 4500 (AB Sciex, Framingham, MA, USA) mass spectrometer in positive ion mode in multiple reaction monitoring (MRM screening) coupled with UHPLC (NEXERA XR, Shimadzu, Tokyo, Japan). Chromatographic separation was achieved on a Synergi 4 µm Fusion-RP 80 A 50 × 2 mm (Phenomenex) column at a flow rate of 0.2 mL/min. The gradient programme was as follows: 20% B (0.3 min), 20–100% B (1.7 min), 100% B (3 min), 100–20% B (0.1 min), 20% B (2 min), solvent A (0.1% formic acid in water: methanol, 95:5 *v*/*v*) and solvent B (methanol with 0.1% formic acid). Column temperature was set to 40 °C. Analyst Software and MultiQuant 3.0.2 (AB, Sciex, Framingham, MA, USA) were used to control the instrument and collect data. The electrospray ionization source was fitted with a stainless-steel capillary (100 μm i.d.). The ion transfer tube temperature was 300 °C. Spray voltage, collision energy, declustering potential and gas parameters were optimized to achieve maximal response using test compounds. Selected reaction monitoring was carried out using the transitions from *m*/*z* 342.92 to 206.00 at 33 eV for VU0152100, *m*/*z* 452.52 to 305.00 at 41 eV for LY 487379, *m*/*z* 446.78 310.00 at 31 eV for internal standard IS VU0152100 and *m*/*z* 469.01 to 319.10 at 31 eV for internal standard IS LY 487379. Calibration curves were constructed, and a linear response was obtained in the range of 0.25 to 500 ng/mL (VU152100) and 1.25 to 2500 ng/mL (LY487379) by spiking known amounts of each compound in blank brain homogenates and plasma. Plasma and brain concentrations of the compounds (in ng/mL) were calculated from calibration curve. Then, brain penetration was calculated as the ratio of the amount of drug in the brain to plasma. 

### 4.6. Statistics

The results were analysed with one-way ANOVA followed by Dunnet’s post hoc comparison or two-way ANOVA followed by Sidak’s post hoc test using GraphPad Prism 7 (GraphPad Software, San Diego CA, USA).

## Figures and Tables

**Table 1:** Plasma or brain concentration (ng/mL) and brain penetration of the highest doses of LY487379 and VU152100 or sub-effective doses of tested compounds when administered in combination (LY487379 + VU152100). Plasma and tissue were collected 30 min after administration. Investigated doses are indicated in parentheses. Data are presented as the means ± SEM. N = 8–10/group. Brain penetration was calculated as the ratio of the amount of drug in the brain to plasma.

	Acute MK-801		Chronic MK-801		Brain/Plasma Ratio	
	Plasma	Brain	Plasma	Brain	Acute MK-801	Chronic MK-801
**Top doses**						
LY 487379 (3 mg/kg)	17.27 ± 1.92	22.23 ± 2.5	21.84 ± 2.8	25.07 ± 3.05	1.28	1.14
VU152100 (1 mg/kg)	101.64 ± 15.51	12.35 ± 1.43	75.46 ± 16.75	7.44 ± 2.67	0.12	0.11
**Sub-effective doses**						
LY 487379 (0.5 mg/kg)	5.3 ± 1.1	5.72 ± 0.79	5.28 ± 0.9	7.30 ± 1.89	1.07	1.38
VU152100 (0.25 mg/kg)	27.05 ± 3.41	4.67 ± 0.46	19.31 ± 3.6	5.66 ± 1.4	0.17	0.29
